# Advance care planning in primary care: a retrospective medical record study among patients with different illness trajectories

**DOI:** 10.1186/s12904-022-00907-6

**Published:** 2022-02-14

**Authors:** Yvonne A. C. Bekker, Ankie F. Suntjens, Y. Engels, H. Schers, Gert P. Westert, A. Stef Groenewoud

**Affiliations:** 1 Radboud University Medical Center, Radboud Institute for Health Sciences, Scientific Center for Quality of Healthcare, PO Box 9101, 6500 HB Nijmegen, the Netherlands; 2 Department of Anesthesiology, Radboud University Medical Center, Pain and Palliative Medicine, Nijmegen, the Netherlands; 3 Department of Primary and Community Care, Radboud University Medical Center, Nijmegen, the Netherlands

**Keywords:** Advance care planning, Primary care, General practice, General practitioner, Medical records, End of life care

## Abstract

**Background:**

Advance Care Planning (ACP) enables physicians to align healthcare with patients’ wishes, reduces burdensome life-prolonging medical interventions, and potentially improves the quality of life of patients in the last phase of life. However, little objective information is available about the extent to which structured ACP conversations are held in general practice.

Our aim was to examine the documentation of ACP for patients with cancer, organ failure and multimorbidity in medical records (as a proxy for ACP application) in Dutch general practice.

**Methods:**

We chose a retrospective medical record study design in seven primary care facilities. Medical records of 119 patients who died non-suddenly (55 cancer, 28 organ failure and 36 multimorbidity) were analysed. Other variables were: general characteristics, data on ACP documentation, correspondence between medical specialist and general practitioner (GP), and healthcare utilization in the last 2 years of life.

**Results:**

In 65% of the records, one or more ACP items were registered by the GP. Most often documented were aspects regarding euthanasia (35%), the preferred place of care and death (29%) and concerns and hopes towards the future (29%). Median timing of the first ACP conversation was 126 days before death (inter-quartile range (IQR) 30–316). ACP was more often documented in patients with cancer (84%) than in those with organ failure (57%) or multimorbidity (42%) (*p* = 0.000). Patients with cancer had the most frequent (median 3 times, IQR 2–5) and extensive (median 5 items, IQR 2–7) ACP consultations.

**Conclusion:**

Documentation of ACP items in medical records by GPs is present, however limited, especially in patients with multimorbidity or organ failure. We recommend more attention for – and documentation of – ACP in daily practice, in order to start anticipatory conversations in time and address the needs of all people living with advanced conditions in primary care.

**Supplementary Information:**

The online version contains supplementary material available at 10.1186/s12904-022-00907-6.

## Key statements

*What is already known:*

- Worldwide, the application of Advance Care Planning (ACP) in daily practice is still low.

- Little is known about the actual practice and documentation of ACP in the GP population, and for different patient groups.

- Previous studies are either self-reporting, or focus on one specific patient group.

*What this paper adds:*

- This paper sheds light on the application and documentation of ACP in medical records of GPs.

- We analysed and contrasted the application of ACP for different illness (and EoL) trajectories (cancer, organ failure, multimorbidity).

- This paper examines the application of ACP in primary care in terms of its contents, timing, extensiveness and frequency.

## Introduction

Advance Care Planning (ACP) is the ongoing process of communication among patients, their healthcare providers, family and loved ones regarding health-related problems, needs and preferences for future care [1, 2]. Timely conversations about these topics enable healthcare providers to align healthcare with the wishes of the patient [3], thereby extending patient autonomy and patient centred care, even when a patient would become unable to participate in decision making due to, for example, cognitive impairment or a crisis situation [4]. ACP is a crucial element of palliative care, which is often indicated when patients suffer from chronic, progressive and life-limiting illnesses [5]. ACP includes items like resuscitation policy, treatment limitations, preferred place of care and death, and conversations about future scenarios [1, 2]. Positive outcomes of ACP have been reported: ACP is found to improve the quality of end of life (EoL) care. It also reduces unwarranted life-sustaining treatments, it prevents hospitalization in the last months of life, and facilitates access to palliative care and hospice care [3, 6–8]. Moreover, it decreases the physical and mental burden of relatives and informal caregivers [7] and it may lower healthcare costs [9].

In many Western countries, *general practitioners* (GPs) play an important role in providing and coordinating palliative care for cancer as well as for non-cancer patients [10]. Also, the long-term relationship between a patient and GP provides a good basis for initiating timely ACP conversations [11]. Therefore, the GP is well positioned to encourage and engage in timely ACP.

However, little is known about the actual application and documentation of ACP in medical records in general practice. Previous studies are mainly based on self-report by GPs [12–15], and documentation has only been studied in a specific GP population of patients with lung and colorectal cancer [16]. Also, patients’ personal wishes, concerns, values and beliefs are often left out, though according to recent definitions and recommendations, they are just as much part of ACP as, for example, specific treatment limitations [1].

Our study’s first *objective* is to provide better insight into the practice and (as a proxy for that) the documentation of ACP in medical records of GPs in the Netherlands. Second, we sought to analyse and contrast if the ACP practice differs for several illness trajectories. We hypothesize that in disease categories with a less predictable trajectory (organ failure, multimorbidity) the prevalence and extensiveness of ACP in general practice will be lower. Finally, we also wanted to see how healthcare utilization at the EoL differed in accordance with differences in the ACP practice.

## Methods

### Study design, setting and data source

For this retrospective medical record study, we retrieved data from the Family Medicine Network (FaMe-net), a primary care registration network in Nijmegen, the Netherlands. This network routinely collects patient data from the electronic GP information system (GPIS) of its seven associated general practices. The database included the medical records of all patients who died between 2003 and 2016 (*n* = 1235). These patient records consisted of: personal characteristics (gender, age at death), reports from the GP, diagnostic ICPC-codes (International Classification of Primary Care), medication prescriptions, correspondence from and to other healthcare providers (of secondary care, out-of-hours primary care and paramedic services) and measured (lab) values, all provided with accompanying dates.

### Study population

First, children (under the age of 18 years) and patients primarily diagnosed with dementia (ICPC-1 P70) were excluded, since both groups form an exception in terms of decisional capacity. Second, a stratified sample of 150 patient records was taken. The number of 150 was chosen, based on relevant literature on the prevalence of disease trajectories at EoL [17]. Besides, we aimed to include at least 30 records per disease category, in order to reach the minimal power for the requested statistical tests. Furthermore, the sample was drawn with an equal distribution among the seven general practices, to reduce the risk of bias, to maintain maximum variation in cases and documentation and to enhance representativity. To compare the application and documentation of ACP in patients with a different trajectory of illness, four groups were created, based on literature: i) patients dying from incurable cancer, whose decline is generally progressive and reasonably predictable, usually with a clear terminal phase [18, 19]; ii) Patients with organ failure (like respiratory and heart failure), whose decline might be punctuated by episodes of acute deterioration and some recovery, with more sudden, seemingly unexpected death;. iii) Elderly with multiple chronic diseases (i.e. multimorbidity) whose decline is often prolonged and gradual [18, 19];. iv) Patients who died suddenly. We used the following inclusion hierarchy: cancer [1], organ failure [2], multimorbidity [3] and acute death [4], meaning that patients who could be classified in more than one category were classified in the first ‘fitting’ category). Allocation was based on medical history and diagnosis was checked and corrected for registration errors. The ‘cancer’ group was defined as patients that had a diagnosed active malignancy at the moment of death (concerning mostly patients with metastasized disease). The ‘organ failure’ group included patients with end-stage heart failure (NYHA 3–4), chronic obstructive pulmonary disease (Gold classification 3–4), renal failure (GFR < 15 ml/min) and symptomatic liver failure. The ‘multimorbidity’ group was defined as patients aged ≥65 years with multimorbidity, meaning ≥2 chronical diseases other than cancer or end-stage organ failure. The ‘acute death’ group included the remaining patients, who died unexpectedly without a history of cancer, organ failure or multimorbidity (e.g. suicide or an acute cardiovascular event). Subsequently, the ‘acute death’ category was excluded, because ACP is less applicable for such patients. Also, files with too limited data for proper group assignment and/or analysis were excluded.

### Data collection

We used patient record information of the last 2 years before death for data extraction. Our rationale was that we expected to find most ACP-related information in the last year(s) of life and that it is recommended to regularly re-discuss ACP themes, especially near the end of life [1]. Also, the widely used surprise question uses a 1-year timeline [20], which we decided to double because we wanted to minimize the risk of missing essential information. We designed a data extraction form to collect quantitative data on patient characteristics and ACP-items (Additional file 1: Appendix 1). In total, 17 ACP-items were defined, based on the recent international consensus on the definition of and recommendations for ACP, supported by the European Association for Palliative Care [1]. A panel discussion with senior investigators (ASG and YE) led to optimization of the form. Then, two researchers (AS and YB) independently collected data from the first nine patient records (three from each group), using the data extraction form. These findings were discussed, which led to final adjustments of the form. No relevant differences between the researchers were found. In this way, inter-observer reliability was considered to be guaranteed, and further in-depth data collection was performed by one researcher (YB).

### Variables

The final data extraction form contained different sections with the following elements (for variables marked with an ^*^, dates (dd/mm/yyyy) were registered):

1. *General characteristics*: patient ID, practice ID, disease category, gender, date of death, age at time of death, place of death, presence of a written euthanasia request and received EoL treatment (palliative sedation, euthanasia, assisted suicide)
2. *Healthcare utilization in the last 2 years of life*: number of hospitalizations^*^, number of emergency department visits^*^ and number of contacts with an after-hours primary care center (telephone contacts, consults at the out-of-hours primary care center and home visits)^*^
3. *ACP items*: documented treatment preferences for future care (regarding resuscitation, mechanical ventilation, intensive care (IC) admission, emergency department referral and hospitalization, antibiotics, artificial feeding and liquid administration and ‘other treatment preferences’), preferred place of care and/or death, registration of advance directives (allocation of a legal representative and a declaration of will) and conversations regarding palliative sedation, euthanasia, prognosis/life expectancy, disease specific future scenarios, personal wishes and goals, concerns and hopes towards the future and the ‘end-of-life’ or death. We define ACP ‘prevalence’ as: at least one documented ACP item (out of these 17 items). ‘Extensiveness’ is the number of documented ACP items. ‘Frequency’ is the documented number of anticipatory conversations regarding ACP items.
4. *ACP characteristics*: ACP presence (≥ 1 item documented), timing of first ACP conversation (documentation; in days until death) and frequency (number of ACP conversations, based on the different dates on which ACP items were reported)^*^

‘First ACP conversation’ and ‘frequency’ (number of ACP conversations) concerned discussions of one or more ACP item(s) between a patient (and his/her family or loved ones) and their GP. ACP items were scored if they consisted of proactive, anticipating discussions, registrations or actions (concerning future treatments or situations). We additionally scored ACP-related information that was found in the correspondence from secondary care medical specialists, out-of-hours GPs and paramedics. ‘Other treatment preferences’, ‘preferences for place of care/death’, ‘personal wishes and goals’ and ‘concerns and hopes towards future’ were extracted as free text for further analysis.

### Data analysis

Statistical analyses were performed using IBM SPSS Statistics 22. We used descriptive statistics for all outcome measures to describe the current ACP practice (first research objective) as well as the health care utilisation (third objective). The extensiveness of ACP was further categorized in ‘no ACP’, ‘1 or 2 items documented’, ‘3-5 items documented’ and ‘6 or more items documented’. Differences between the three disease categories (second research objective) were assessed using Pearson’s chi-square test or Fischer’s exact test (in case > 20% of the cells had an expected count less than 5) for categorical variables. For continuous variables, we used the ANOVA in case the distribution was normal and the Kruskal Wallis test in case the distribution was skewed. A *P* value of < 0.05 was considered significant. In case of significance, we performed post hoc analysis, comparing groups mutually (pairwise) using independent t-tests (or Mann-Whitney U) and Pearson’s chi-square tests (or Fisher’s exact). A Bonferroni correction was applied, adjusting the level of significance to correct for multiple comparisons [21]. This led to a significance level of *p* < 0.0167 (0.05/3). Qualitative thematic analysis was applied on the extracted free text that had been entered in the files by GPs. We applied an open coding technique [22] that was simultaneously performed by two researchers (YB and AS), who discussed codes together and reached consensus on both codes and themes. Emerging themes were used to illustrate and underpin results.

## Results

### Study sample

**Fig. 1:**
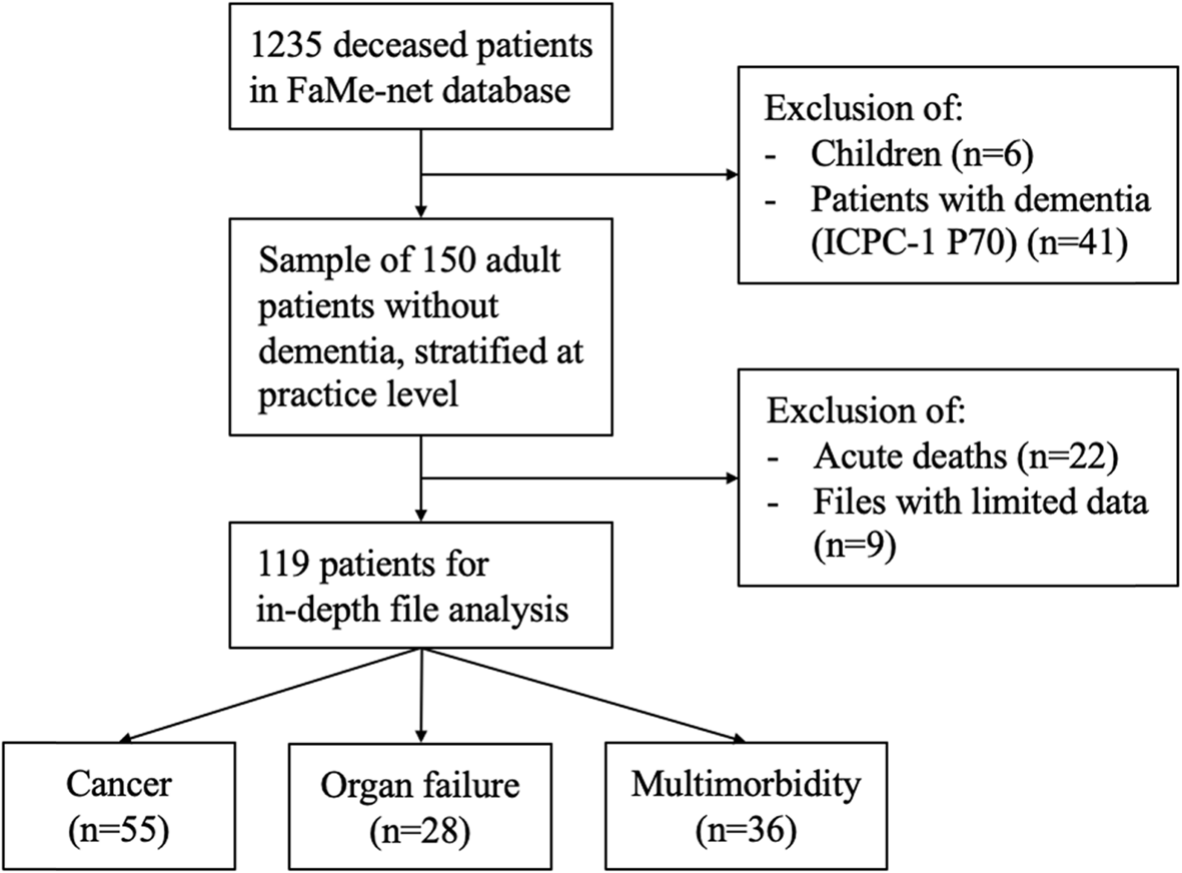
Selection of patients who died non-suddenly of ‘cancer’, ‘organ failure’ or ‘multimorbidity’ in general practice

After the selection of 150 records, and applying the exclusion criteria, medical records of 119 patients who died non-suddenly were identified for the in-depth file research, of whom 55 patients with cancer, 28 with organ failure and 36 with multimorbidity (see flowchart in Fig. 1).

### Patient characteristics and general information about the last 2 years of life

The mean age of the patients at time of death was 79 years (range: 27–103; see Table 1). Patients with cancer were younger than patients with organ failure and multimorbidity (both *p* = 0.000). The male:female ratio was approximately 1:1 for all groups. Most patients died at home (59%). Other places of death were hospital (27%), hospice (5%), and long-term care facility (3%).

**Table 1:** Patient characteristics and healthcare use in the last two years of life (subgroup analyses for cancer, organ failure and multimorbidity patients)

General characteristics	Total (*n* = 119)	Cancer (*n* = 55)	Organ failure (*n* = 28)	Multimorbidity (*n* = 36)	*P* value
Gender female, n (%)	61 (51)	29 (53)	14 (50)	18 (50)	0.957^*^
Age at time of death, mean (SD)	79 (14)	70 (14)	84 (8)	88 (7)	0.000^**^
Place of death, n (%)					0.088^***^
Home	70 (59)	35 (64)	16 (57)	19 (53)	
Hospital	32 (27)	11 (20)	7 (25)	14 (39)	
Hospice	6 (5)	6 (11)	0 (0)	0 (0)	
Long-term care facility	4 (3)	1 (2)	1 (4)	2 (6)	
Unknown	7 (6)	2 (4)	4 (14)	1 (3)	
EoL support, n (%)					
Palliative sedation	32 (27)	22 (40)	2 (7)	8 (22)	0.005^*^
Euthanasia/assisted suicide	5 (4)	5 (9)	0 (0)	0 (0)	0.085^***^
Healthcare use, median (25th–75th percentile, range)					
Number of hospitalizations	1 (0–3, 0–15)	2 (1–3, 0–11)	2 (1–4, 0–15)	0,5 (0–1, 0–4)	0.000^**^
Number of ED visits	1 (0–2, 0–9)	1 (0–3, 0–6)	2 (1–3, 0–9)	1 (0–1, 0–6)	0.006^**^
Number of GP out of office hours contacts	1 (0–4, 0–22)	1 (0–4, 0–22)	2 (0–5, 0–6)	1 (0–2, 0–8)	0.316^**^

^*^ Chi Square test

^**^ Kruskal Wallis test

^***^ Fisher’s Exact test

^****^ Mann Whitney U test

All patients who died in a hospice were patients with cancer. Most patients who died in a hospital had multimorbidity (44%) or cancer (34%). Prevalence of (intermittent or continuous) palliative sedation was highest in the cancer group (40%), followed by the multimorbidity (22%) and the organ failure group (7%). Ten patients (8%) had a documented written euthanasia directive. Euthanasia was actually performed in 50% of those patients, who all had cancer. More than two thirds of the patients visited the emergency department (70%) and were hospitalized (72%) at least once in the last 2 years of life. The median numbers of hospitalizations, emergency department visits, and out-of-hours primary care contacts are shown in Table 1.

### Application of ACP

Prevalence of ACP (≥ 1 item documented) by the GP in the last 2 years of life was 65%. In 40% of all medical records, three or more items were registered, and in 18% six or more items. The prevalence differed significantly between the three groups, with respectively 84, 57 and 42% in patients with cancer, organ failure and multimorbidity (*p* = 0.000) (see Table 2). Post hoc analyses showed that ACP was applied more often in the cancer group than in the organ failure and multimorbidity group (*p* = 0.015 and p = 0.000 respectively). There was no statistically significant difference between the organ failure and multimorbidity group (*p* = 0.314). The prevalence of ACP also differed between practices (*p* = 0.030), as some of the practices had a higher prevalence than others (range 42–83%).

**Table 2:** Prevalence and characteristics of Advance Care Planning by GP (subgroup analyses for cancer, organ failure and multimorbidity patients)

Prevalence of ACP (documentation)	Total (*n* = 119)	Cancer (*n* = 55)	Organ failure (*n* = 28)	Multimorbidity (*n* = 36)	*P* value
Prevalence (≥ 1 ACP item), n (%)	77 (65)	46 (84)	16 (57)	15 (42)	0.000^*^
**Characteristics of ACP**	Total (*n* = 77)	Cancer (*n* = 46)	Organ failure (*n* = 16)	Multimorbidity (*n* = 15)	*P* value
Extent					
Number of ACP items, median (25th -75th percentile, range)	4 (2–7, 1–11)	5 (2–7, 1–11)	3,5 (2–6, 1–8)	2 (1–5, 1–7)	0.047^**^
Subclassification:					0.222^***^
Low (1 or 2 items)	29 (38)	15 (33)	6 (38)	8 (53)	
Medium (3, 4 or 5 items)	26 (34)	14 (30)	6 (38)	6 (40)	
High (≥ 6 ACP items)	22 (29)	17 (37)	4 (25)	1 (7)	
Frequency					
Number of ACP conversations, median (25th–75th percentile, range)	3 (1–4, 1–14)	3 (2–5, 1–14)	2 (1–3, 1–7)	2 (1–3, 1–4)	0.012^**^
Timing					
Time between first ACP conversation and death in days, median (25th–75th percentile, range)	126 (30–316, 1–714)	106 (22–307, 3–680)	227 (39–395, 1–714)	113 (52–320, 10–529)	0.417^**^

* Chi Square test, ** Kruskal Wallis test, *** Fisher’s Exact test

If ACP was applied (at least one item documented), the frequency (median number of ACP conversations) was three, with a median of four different items that were documented. The extent (number of ACP items documented) and frequency (number of ACP conversations) of ACP differed significantly between the groups (*p* = 0.047 and *p* = 0.012, respectively). Post hoc analyses (Table 3) showed that the number of consultations in which ACP was documented was significantly higher in patients with cancer than in patients with multimorbidity (*p* = 0.010). All other post hoc analyses did not show a statistically significant difference. The median time period from the first ACP documentation until death was 126 days (interquartile range (IQR) 30–316), with no significant difference between patient groups. IQRs showed a large variation in timing in all three disease groups.

**Table 3:** Post-hoc tests on differences between groups of patients

***P*** values of post-hoc analyses	Cancer vs Organ failure	Cancer vs multimorbidity	Organ failure vs multimorbidity
Prevalence (≥ 1 ACP item documented)	0.015^*^	0.000^*^	0.314^*^
Extent (number of ACP items)	0.417****	0.023^****^	0.175^****^
Frequency (number of ACP conversations)	0.043^****^	0.010^****^	0.520^****^

^*^ Chi Square test

^****^ Mann Whitney U test

**Fig. 2:**
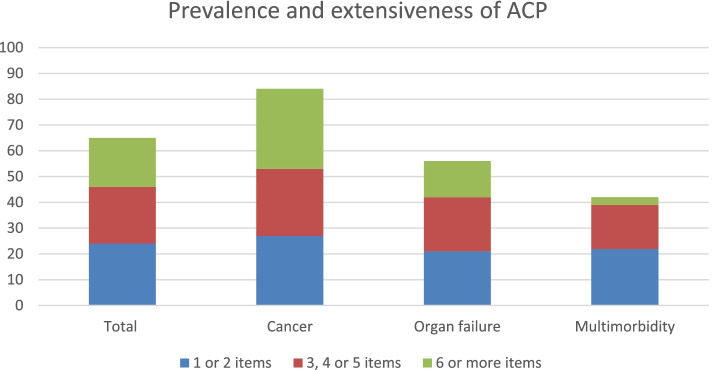
Prevalence and extensiveness of ACP (number of ACP items documented) for all patients and sub-groups, in percentages

The prevalence of ACP for all patients and for the different disease categories is visualized in Fig. 2. The colours in the bars represent the number of ACP items discussed.

### Content of ACP

If aspects of ACP were documented by a GP, this most often regarded: euthanasia (35%), preferred place of care and death (29%), concerns and hopes towards the future (29%), prognosis (24%) and palliative sedation (23%) (see Table 4).

**Table 4:** ACP content as documented by GPs (subgroup analyses for cancer, organ failure and multimorbidity patients)

Items of ACP	Total (*n* = 119)	Cancer (*n* = 55)	Organ failure (*n* = 28)	Multimorbidity (*n* = 36)	*P* value
Documented treatment preferences for future care, n (%)					
Resuscitation	25 (21)	11 (20)	11 (39)	3 (8)	0.009
Mechanical ventilation	2 (2)	2 (4)	0 (0)	0 (0)	0.499
Intensive Care Unit admission	4 (3)	2 (4)	2 (7)	0 (0)	0.266
Referral to ED and hospitalization	22 (19)	8 (15)	8 (29)	6 (17)	0.286
Antibiotics	4 (3)	2 (4)	1 (4)	1 (3)	1.000
Artificial feeding and liquid administration	1 (1)	1 (2)	0 (0)	0 (0)	1.000
Other treatment preferences	18 (15)	11 (20)	4 (14)	3 (8)	0.308
Documented EoL wishes, n (%)					
Preferred place of care/death	35 (29)	20 (36)	7 (25)	8 (22)	0.327
Personal wishes/goals	18 (15)	16 (29)	1 (4)	1 (3)	0.000
Discussed future scenarios, n (%)					
Prognosis/life expectancy	29 (24)	18 (33)	8 (29)	3 (8)	0.024
Disease specific future scenarios	20 (17)	16 (29)	3 (11)	1 (3)	0.002
Concerns and hopes towards future	34 (29)	20 (36)	7 (25)	7 (19)	0.206
Conversation about palliative sedation	27 (23)	22 (40)	4 (14)	1 (3)	0.000
Conversation about euthanasia	42 (35)	35 (64)	2 (7)	5 (14)	0.000
Conversation ‘end-of-life’	22 (19)	14 (26)	5 (18)	3 (8)	0.136
Registered advance directives, n (%)					
Declaration of will	19 (16)	18 (33)	0 (0)	1 (3)	0.000
Appointment of legal representative	0 (0)	0 (0)	0 (0)	0 (0)	–

Most often documented treatment preferences were resuscitation policy (21%) and policy for referral and hospitalization (19%). ‘Other treatment preferences’ consisted mostly of disease specific treatment choices for future care, such as chemotherapy, follow-up diagnostics, surgical procedures and medication choices or limitations (other than antibiotics). The items ‘personal wishes and goals’ and ‘hopes and concerns towards the future’ mainly concerned themes related to the different domains of palliative care, i.e. psychological, social and spiritual issues. Examples of personal wishes and goals were: attending specific future events (like the graduation of a grandson or a holiday with family); spending more time outside in the last months of life; regaining enough energy to receive visitors; being able to carry out personal hobbies (like gardening or etching) and the wish for conversations with a chaplain or pastor. Regarding hopes and concerns towards the future, patients mainly expressed their fears, such as cognitive impairment, being a burden to others, being left alone or death itself. Patients hoped, for example, for passing away naturally in the short term. In 19% of the cases, it was documented that the GP and patient had had a conversation about the ‘end-of-life’, without further documenting an outcome or specific preferences. When going through the patient records, we noticed that GPs often used loose terms when documenting about ACP, for example: ‘conversation about death’, ‘best supportive care’, ‘palliative policy’ or ‘prefers quality instead of quantity’. Also, place of care and death were mostly unseparated, and some patients did not specify their preference, but only discussed which options they did not prefer. Registration of a declaration of will was present in 8% of the cases. The appointment of a legal representative was never documented.

### Impact of ACP on actual care (utilisation)

Discussion of particular aspects of ACP seems to be associated with the actual (utilisation of) care. When documented (29%), the preferred place of care/death was ‘home’ in 57% and ‘hospice’ in 26% of the cases. If GPs discussed and documented the patient’s preferred place of care/death, more patients died in a hospice (*p* = 0.010). When the GP and patient had at least one conversation about palliative sedation, more patients were supported by palliative sedation in the last days of life (*p* = 0.000). Documentation of a policy for referral and hospitalization did not influence the number of Emergency Room (ER) visits (*p* = 0.838) and hospitalizations (*p* = 0.793).

### Exchanging ACP information with other health care professionals

GPs also received ACP-related information from other healthcare providers (see Table 5). In 50% of the medical records, at least one ACP item was found in the correspondence between other healthcare providers and GPs. This concerned mainly resuscitation (28%) and Intensive Care Unit (ICU) admission (17%) policies and discussion of prognosis/life expectancy (21%). All other items had a lower prevalence (0–11%).

**Table 5:** Content of ACP in correspondence of other healthcare provider (subgroup analyses for cancer, organ failure and multimorbidity patients)

Items of ACP	Total (*n* = 119)	Cancer (*n* = 55)	Organ failure (*n* = 28)	Multimorbidity (*n* = 36)	*P* value
Documented treatment preferences for future care, n (%)					
Resuscitation	33 (28)	16 (29)	11 (39)	6 (17)	0.129
Mechanical ventilation	9 (8)	7 (13)	1 (4)	1 (3)	0.150
Intensive Care Unit admission	20 (17)	9 (16)	9 (32)	2 (6)	0.019
Referral to ED and hospitalization	7 (6)	3 (6)	3 (11)	1 (3)	0.470
Antibiotics	0 (0)	0 (0)	0 (0)	0 (0)	–
Artificial feeding and liquid administration	1 (1)	1 (2)	0 (0)	0 (0)	1.000
Other treatment preferences	13 (11)	9 (16)	3 (11)	1 (3)	0.119
Documented EoL wishes, n (%)					
Preferred place of care/death	11 (9)	8 (15)	1 (4)	2 (6)	0.272
Personal wishes/goals	5 (4)	4 (7)	1 (4)	0 (0)	0.199
Discussed future scenarios, n (%)					
Prognosis/life expectancy	25 (21)	21 (38)	3 (11)	1 (3)	0.000
Disease specific future scenarios	13 (11)	11 (20)	1 (4)	1 (3)	0.017
Concerns and hopes towards future	5 (4)	4 (7)	0 (0)	1 (3)	0.439
Conversation about palliative sedation	4 (3)	3 (6)	1 (4)	0 (0)	0.358
Conversation about euthanasia	8 (7)	6 (11)	1 (4)	1 (3)	0.319
Conversation ‘end-of-life’	2 (2)	2 (4)	0 (0)	0 (0)	0.499

In both the GP and the other healthcare provider documentation, all of the ACP topics showed the highest prevalence rate in the ‘cancer’ group, with exception of the resuscitation, hospital referral and ICU-admission policy, which were documented most frequently in the ‘organ failure’ group.

In 24% of the patients, no ACP items were found in both the GP’s file and the correspondence of other healthcare providers.

## Discussion

We found a prevalence of documented ACP activities during the last 2 years of life in GPs’ medical records of patients with cancer, organ failure or multimorbidity of 65%. However, the extensiveness of documented ACP was limited, as in 71% five or less ACP items were covered, and in 38% only one or two (out of 17). Concluding that, in the total population, in the majority of patients ACP was not or only minimally (one or two items) documented (71/119, 60%). For most patients who received ACP, the first ACP conversation took place during the last months of life (median 126 days before death) and the median frequency (number of ACP conversations) was three. Documented ACP mainly focused on euthanasia, concerns and hopes towards the future and the preferred place of care and death. Remarkably, a resuscitation policy was noted by the GP in only 21%, and other treatment preferences and disease specific future scenarios were documented even less, while these are topics that play an important role in case of acute deterioration.

In half of the patients, GPs also received ACP information through the correspondence from other healthcare providers. In such cases, the main ACP aspects concerned resuscitation policy (28%), prognosis (21%) and ICU admission (17%). Other treatment decisions that take place in the hospital context (such as (dis)continuation of chemotherapy) were scarcely found. In 24% of the patients, no ACP items were found in both the GPs’ file and the correspondence of other healthcare providers.

Application and documentation of ACP differed significantly between the disease categories. The prevalence of ACP was higher in patients with cancer (84%) than in those with organ failure (57%) or multimorbidity (42%). We also found statistically significant differences between the disease categories regarding the number of ACP-items and consultations in which ACP was discussed, with cancer rating highest on both aspects.

### Strengths and limitations

To the best of our knowledge, this was the first study that seeked to explore actual ACP documentation in the population of non-acute deaths in the Dutch general practice, while discerning between different illness (and EoL) trajectories. We picked up on the recent shift in extending ACP also to non-physical issues, as physical changes are usually accompanied by psychological, social and existential fluctuations in the well-being of patients and their families, seen in both cancer as well as non-cancer patients [23]. We were able to define 17 ACP items, covering the physical, psychosocial and spiritual dimension, based on the consensus definition and recommendations for ACP supported by the European Association for Palliative Care and expert opinion. By taking a stratified sample that equally represents seven different practices, we enhanced the representativity of our results and minimized the influence of between-practice differences on the found group differences. FaMe-net also aims to be representative of the national population by age, gender and cause of death. Last, our study protocol comprises several check-points to enhance inter-observer reliability (reproducibility).

Also, some *limitations* must be acknowledged. First, scanned documents, attached to the medical record, were missing from the database, which might explain the low percentage of advance directives and the absence of information on the appointment of legal representatives. Second, data were collected in general practices from a practice-based research network, which are known to have a better registration routine than average. This may have resulted in an overestimation of ACP documentation. On the other hand, participating practices were not specialized in palliative care, and no specific codes for ACP registration are used in the network. Third, there is a risk of missing ACP related information, as ACP discussions might have taken place without documentation. However, registration of ACP content is essential to facilitate future decision-making that is based on a patient’s preferences and is, therefore, a condition to successful ACP, especially since in most health care settings multiple caregivers are involved. Fourth, we may have also missed information by only looking at the last 2 years of life. In our opinion, though, information dating from longer than this period is less relevant as patients’ preferences and wishes possibly change along with changes in their medical condition [24]. This is why ACP should be frequently re-discussed [1]. Lastly, our sample contains files from a broad time period up until 2016. Although the majority of the files (80/119) dates from the most recent period (2014–2016), this prohibits us from drawing firm conclusions about the current situation.

### Interpretation

Our finding that 35% of the deceased patients seemed *not* to have received any form of ACP consultation by their GP and 60% not or only minimally (one or two items documented), is in line with existing literature about the uptake of ACP in general practice, and may be explained by some barriers for ACP that have been reported before (see Table 6). Recently, patient records in the general practice were examined in Scotland, showing that 60% of all patients who died in 2014 had an anticipatory care plan; 75% of the patients with cancer and 41% with organ failure [25]. Though this was a study in a different country, these percentages are quite comparable with our findings. Ermers et al. found that, in the Netherlands, in 74% of the records of patients with colorectal and lung cancer, at least one ACP item was documented, compared to 84% in our group of patients with an active malignancy at the moment of death [16].

**Table 6:** Barriers for the uptake of ACP, as reported in the literature

Despite the fact that there is increasing attention for ACP and a growing body of evidence of its positive effects, research shows that the application of ACP conversations remains still low, and the organization and delivery of healthcare is still predominantly reactive [11–16]. Several barriers have been reported that may prevent optimal implementation in clinical practice. First, on the patient-side, participation is at risk in case patients are not ready to talk about themes related to deterioration in their condition or the nearing death [41]. Second, for GPs, insufficient time is among the most important barriers. Third, GPs find it difficult to engage in end-of-life conversations, which is sometimes caused by lack of skills or experience, and that they have a hard time finding the appropriate moment to initiate ACP [27, 31, 33, 42]. Fourth, illness trajectories differ a lot from patient (group) to patient (group). In patients with incurable cancer, the decline is generally progressive and reasonably predictable, usually with a clear terminal phase [18, 19]. Patients dying from a non-malignant cause, however, frequently experience a more gradual decline. In those with organ failure (like respiratory and heart failure), the decline might be punctuated by episodes of acute deterioration and some recovery, with more sudden, seemingly unexpected death. In the elderly with multiple chronic diseases (i.e. multimorbidity) the decline is often prolonged and gradual [18, 19]. Especially in case of uncertainty of prognosis, there are less clear-cut ‘triggers’ that may help GPs to initiate ACP conversations [31].	

Second, our finding that ACP is more prevalent among cancer patients, is in line with the results of other studies [12–14, 25, 26]. Although these studies are based on questionnaires and interviews, whereas our study has the design of a medical record study, existing literature strengthens our conclusion that patients with organ failure and multimorbidity receive less ACP. This might be due to prognostic uncertainty; the relative unpredictability of non-cancer patients’ decline possibly impedes physicians to anticipate palliative care needs and initiate end-of-life conversations timely [27]. As there are less direct causes to start the conversations, GPs might prevaricate or postpone when considering end-of-life issues, also described in literature as prognostic paralysis [28]. Also, the need for ACP is often less clear when there is no strict demarcation between the curative and the palliative phase [29]. Patients suffering from cancer are more aware of the life-threatening consequences of their disease and engage in ACP more proactively [30], while patients still being treated in the hospital are less open to ACP discussions, as was reported by GPs in earlier research [31]. Nonetheless, ACP is also appropriate alongside optimal chronic disease management and has been widely recommended for non-cancer patients [1, 14, 20, 32]. GPs, however, appear to find this difficult to implement [20, 27, 29, 31–34], resulting in non-cancer patients being relatively underserved with regard to comprehensive and timely ACP.

Third, our results support earlier findings that ACP is initiated late in the disease process; other studies found a timing of ACP that ranged from 33 days until 18 weeks before death, compared to 126 days before death in our study [16, 25, 29, 35].

### Implications and recommendations

Though ACP receives more and more attention, widespread implementation in clinical practice stays behind. A higher prevalence of ACP, broader discussion of ACP-related themes and timely initiation could benefit the quality of end-of-life care. It appears desirable to close the gap between patients with and without cancer and offer ACP to all patient groups, especially when considering the growing number of old people suffering and dying from serious chronic diseases [36]. Extending an anticipatory care approach to all people with advanced chronic conditions is a challenge and multiple barriers (see Table 6) need to be overcome. Professionals caring for people with life-limiting conditions need core generic skills to enable them to assess supportive care needs and judge the readiness of individual patients and families to participate in discussions about the future. However, many physicians feel poorly prepared to conduct end-of-life conversations [37]. Recently, in the Netherlands, ACP training programs have been developed, such as the course ‘Timely end of life conversations’ by the Dutch GP Association and the RADPAC training [34]. These trainings might serve as useful aids to educate physicians and to help them deal with feelings of uncertainty, though they are not widely implemented yet. Also, GPs have been appointed as key-players in ACP in the recently launched Quality Framework for Palliative Care in the Netherlands, which may also further improve the uptake of ACP in primary care [38]. Another starting point for improvement is the structure of documentation. Our study shows that there is no uniform structure in registering ACP information in the Netherlands, and we found that both GPs and medical specialists used heterogenous and a specific terminology, which makes it hard to retrieve, transfer and update information. This could be improved by using predictable, homogeneous and exchangeable formats to document ACP, which has been shown to be successful in other countries [25, 39]. Also, a distinction should be made between preferred place of care and preferred place of death, as these do not always have the same outcome [40]. Lastly, timely recognition of patients that could benefit from ACP is essential, but appears to be difficult. In the group of organ failure, hospital admissions and exacerbations might serve as a helpful starting points for discussion about wishes and needs [23, 31]. We recommend that future research contributes to the identification of patients that may benefit from ACP, as well as the appropriate timing to initiate ACP conversations.

## Conclusion

This study shows that at least one ACP -item is documented in the medical record in a majority of deceased patients in the Dutch general practice. However, there is considerable potential for improvement in the documentation (and practice) of ACP, concerning the amount of topics covered, disease specific future scenarios and treatment preferences, timely initiation, and the documentation structure and multidisciplinary information exchange. Also, attention is needed for the current gap between patients with cancer and patients with other chronic diseases, addressing the needs of all people living with advanced conditions in primary care.

## Supplementary Information

**Additional file 1: Appendix 1**. Data extraction form.

## Data Availability

The datasets generated during and analyzed during the current study are not publicly available for reasons of privacy. They are however available (fully anonymised) from the corresponding author on reasonable request.

## Abbreviations

ACP: Advance Care Planning
EoL: End of Life
ED: Emergency Department
GP: General Practitioner
GPIS: General Practitioner’s Information System
ICU: Intensive Care Unit
RADPAC: RADboud indicators for PAlliative Care
