# Supplementary material for: Advance care planning in primary care: a retrospective medical record study among patients with different illness trajectories
Source: BMC Palliat Care. 2022 Feb 14;21:21. doi: 10.1186/s12904-022-00907-6 (PMC8842525; doi:10.1186/s12904-022-00907-6)
Supplement: Supplementary file 1 — Additional file 1: Appendix 1. Data extraction form. [file 12904_2022_907_MOESM1_ESM.docx]

**Appendix 1: Data extraction form**

**Patient characteristics**

| Patient ID |  |
| --- | --- |
| Practice ID |  |
| Subgroup | - Cancer □ Organ failure □ Multimorbidity |
| Gender | - Male □ Female |
| Date of death |  |
| Age at time of death | - Hospice - Other - Unknown |
| Place of death | - Home - Long-term care facility - Hospital |
| EoL treatment | - Palliative sedation - Euthanasia/assisted suicide |
| Euthanasia request | - Present |
| Number of hospitalizations (+dates) | # |
| Number of emergency department visits (+dates) | # |
| Number of contacts with after-hours primary care center (+dates) | # |

**Advance Care Planning**

| Date of first ACP conversation |  | |
| --- | --- | --- |
| Number of ACP conversations |  | |
| Documented treatment preferences for future care | CPR policy | □ GP  □ Other healthcare provider |
|  | Mechanical  ventilation | □ GP  □ Other healthcare provider |
|  | IC policy | □ GP  □ Other healthcare provider |
|  | Hospital admissions/  emergency department | □ GP  □ Other healthcare provider |
|  | Antibiotics | □ GP  □ Other healthcare provider |
|  | Artificial feeding and  liquid administration | □ GP  □ Other healthcare provider |
|  | Other  (i.e. chemotherapy, radiation, surgery ,  pacemaker, ICD, dialysis, oxygen therapy,  medication, etc..)  Namely: | □ GP  □ Other healthcare provider  ………………………………………………… |
| Documented EoL wishes | Preferred place of care/death  Namely: | □ GP  □ Other healthcare provider  ……………………………………………….. |
|  | Personal wishes/goals | □ GP  □ Other healthcare provider |
| Discussed future scenarios | Prognosis/life expectancy | □ GP  □ Other healthcare provider |
|  | Disease specific future scenarios | □ GP  □ Other healthcare provider |
|  | Concerns and hope towards future | □ GP  □ Other healthcare provider |
|  | Conversation about palliative sedation | □ GP  □ Other healthcare provider |
|  | Conversation about euthanasia | □ GP  □ Other healthcare provider |
|  | Conversation about ‘end-of-life’/death | □ GP  □ Other healthcare provider |
| Registered advance directives | Allocation of legal representative | □ GP  □ Other healthcare provider |
|  | Declaration of will | □ GP  □ Other healthcare provider |
